# MicroRNAs: Pleiotropic Regulators in the Tumor Microenvironment

**DOI:** 10.3389/fimmu.2018.02491

**Published:** 2018-11-01

**Authors:** Ning Yang, Shan Zhu, Xinping Lv, Yuan Qiao, Yong-Jun Liu, Jingtao Chen

**Affiliations:** ^1^Institute of Translational Medicine, The First Hospital of Jilin University, Changchun, China; ^2^Sanofi Research and Development, Cambridge, MA, United States

**Keywords:** microRNAs, immune cells, cancer-associated endothelial cells, cancer-associated fibroblasts, tumor cells, crosstalk, tumor microenvironment

## Abstract

MicroRNAs (miRNAs) are small non-coding RNAs that typically inhibit the translation and stability of messenger RNAs (mRNAs). They are ~22 nucleotides long and control both physiological and pathological processes. Altered expression of miRNAs is often associated with human diseases. Thus, miRNAs have become important therapeutic targets, and some clinical trials investigating the effect of miRNA-based therapeutics in different types of diseases have already been conducted. The tumor microenvironment (TME) comprises cells such as infiltrated immune cells, cancer-associated endothelial cells (CAEs) and cancer-associated fibroblasts (CAFs), and all the components participate in the complicated crosstalk with tumor cells to affect tumor progression. Altered miRNAs expression in both these stromal and tumor cells could drive tumorigenesis. Thus, in this review, we discuss how aberrantly expressed miRNAs influence tumor progression; summarize the crosstalk between infiltrated immune cells, CAEs, CAFs, and tumor cells through miRNAs, and clarify the important roles of miRNAs in the tumor microenvironment, which may facilitate the clinical application of miRNA-based therapies.

## Introduction

MicroRNAs (miRNAs) are functional single-strand RNAs, ~22 nucleotides long, which are transcribed in the nucleus first by RNA polymerase II, called pri-miRNAs. After they are cleaved by Drosha and DGCR8, pri-miRNAs are transferred to pre-miRNAs, and then with the aid of exportin 5, pre-miRNAs were transported to the cytoplasm and processed by Dicer, leading to the formation of double-stranded microRNA, and then the dimeric RNAs are unwound by Argonaute proteins and incorporated into RNA-induced silencing complex (RISC). RISC directly binds to the 3′ untranslated region (UTR) of targeted mRNAs to degrade or repress translation (1).

The first miRNA was identified in *Caenorhabditis elegans* by Lee et al. (2), who found that a short RNA product encoded by *lin-4* could partially complement the 3′ UTR of *lin-14* mRNA, reduce the amount of lin-14 protein, and regulate the development of *C. elegans*. Following the discovery of the second miRNA, *let-7*, which is conserved in many organisms, the roles of miRNAs in non-nematode species became apparent (3). To date, miRNAs have been found to participate in many physiological processes such as cell cycle regulation, proliferation, apoptosis, and neurogenesis (4). miRNAs regulate up to 60% of protein-coding genes (5), and the deregulation of miRNAs is associated with various human diseases, including cancer, autoimmune diseases, cardiovascular and neurological disorders (6).

In tumor cells, aberrantly expressed miRNAs exert tumor-suppressive or oncogenic functions by regulating the expression of mRNAs in different signaling pathways, thereby affecting tumor progression (7). The miR-15 and miR-16 were the first miRNAs linked to cancer. In 2002, Calin et al. found they were downregulated in most patients with chronic lymphocytic leukemia (CLL) (8). Furthermore, miR-155, miR-21, and miR-210 expression levels are elevated in the sera of patients with diffuse large B-cell lymphoma (DLBCL) (9). These observations suggested the use of miRNAs as diagnostic and prognostic biomarkers. miRNAs are also regarded as therapeutic targets in human cancer, and several related clinical trials have been conducted (10).

The tumor microenvironment (TME) is very complex and in addition to tumor cells, comprises several infiltrated cell types such as infiltrated immune cells, cancer-associated endothelial cells (CAEs), and cancer-associated fibroblasts (CAFs). These cells significantly contribute to tumor progression, and deregulated miRNAs expression in these cells might determine the fate of the tumor. In this review, we summarize the role of miRNAs in the crosstalk between these cells and tumor cells, to enhance our understanding of the significance of miRNAs in the TME and lay a foundation for miRNA-based therapies in cancer treatment.

## miRNAs play a role in the modulation of the TME

### miRNAs as modulators between infiltrated immune cells and tumor cells

Different types of infiltrated immune cells are involved in the TME and contribute to the regulation of the fine balance between anti- and pro-tumor signals. Among them, lymphocytes (especially T cells), natural killer cells (NKs), and dendritic cells (DCs) are crucial for tumor suppression, while regulatory T cells (Tregs), myeloid-derived suppressor cells (MDSCs), and tumor-associated macrophages (TAMs) are considered to play immunosuppressive roles. As multifaceted regulators, miRNAs influence the differentiation and function of the immune cells mentioned above. The miR-155, for example, has been reported to be widely expressed in immune cells. In T cells, it was initially found to regulate the Th cell lineage decision, because miR-155-deficient mice display a bias toward Th2 differentiation (11). Tregs proliferation and homeostasis can also be modulated by miR-155 (12). In NKs, miR-155 was found to promote NK expansion and functional activation and enhance the production of IFN-γ (13, 14). miR-155 deficiency in DCs impairs their maturation, migration ability, cytokine production, and ability to activate T cells (15). In MDSCs, miR-155 is involved in the expansion of MDSCs and is required to facilitate tumor growth (16). In TAMs, the downregulation of miR-155 increases the production of IL-10, enhancing its immunosuppressive function (17). The roles of miRNAs in immune and cancer cells have been thoroughly reviewed elsewhere (7, 18), while the miRNA-mediated crosstalk between tumor-infiltrated immune cells and tumor cells in the TME remains to be elucidated. Hence, in this section, we will discuss several representative and well-studied miRNAs that act as critical modulators of the immune response in the TME.

#### miRNAs as modulators between T cells and tumor cells

T lymphocyte-mediated immune responses are critical for tumor immunity. After activation, CD4^+^ T cells differentiate into different types of T helper cells (Th1, Th2, Th17, and Tfh) and Tregs. miRNAs have been increasingly recognized as important modulators of Th cells and Tregs fate decisions and effector functions (19). Th2 and Th17 cells mainly mediate responses against helminths, bacteria, and fungi, and although they are necessary for effective T cell-dependent antibody responses, they do not participate directly in tumor immunity. Thus, the miRNA-mediated crosstalk with these cells will not be discussed here.

**Figure 1:**
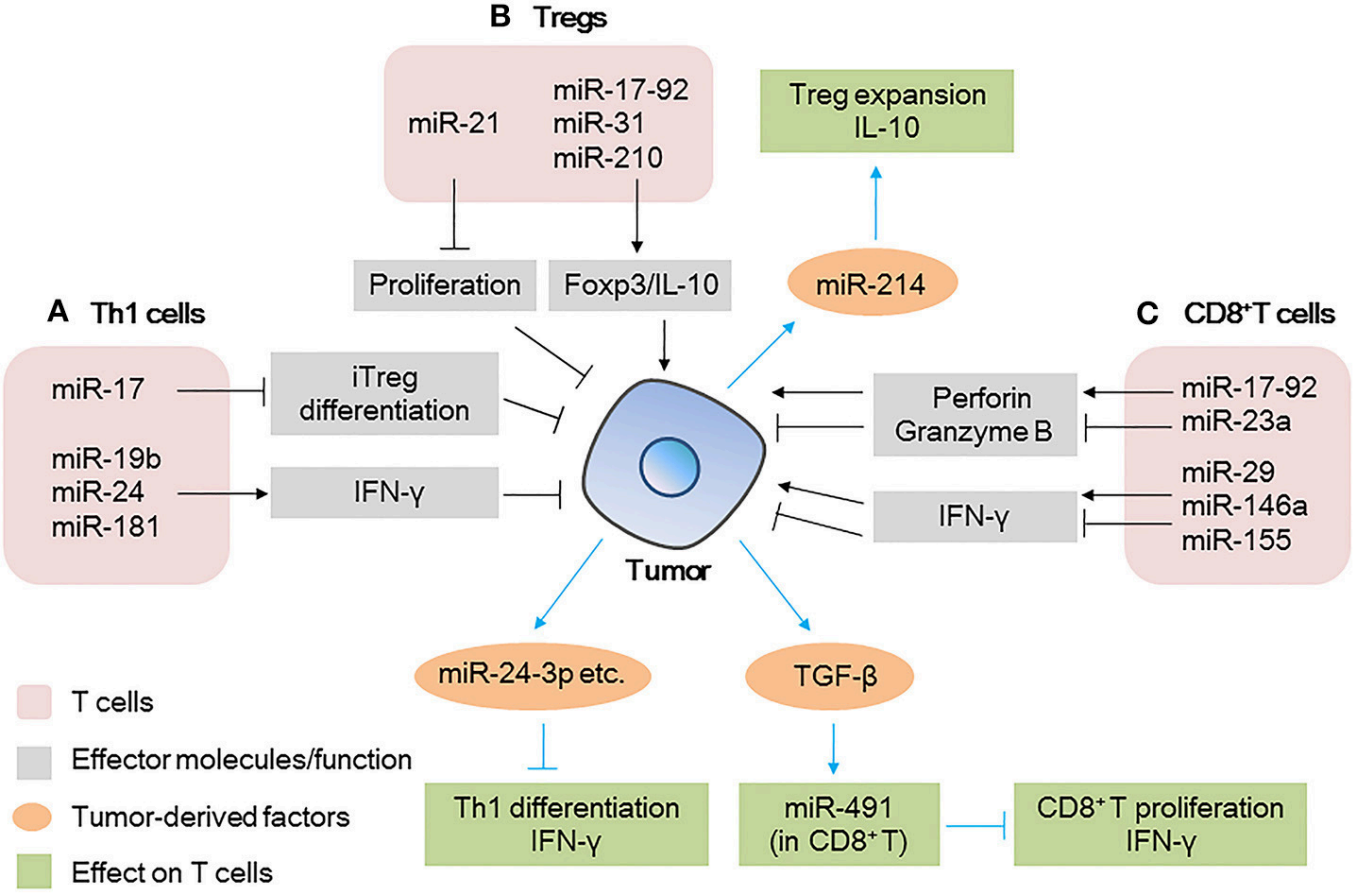
MicroRNAs (miRNAs) act as modulators between T cells and tumor cells **(A)** miRNAs expressed in Th1 cells modulate tumor progression by inducing iTreg differentiation or secreting IFN-γ; tumor-derived miRNAs affect the differentiation/IFN-γ production by Th1 cells. **(B)** miRNAs expressed in Tregs modulate tumor progression by regulating transcription factor expression or cytokine production; tumor-derived miRNAs affect the expansion/cytokine production in Tregs. **(C)** miRNAs expressed in CD8^+^ T cells modulate tumor progression by regulating effector molecule (IFN-γ and perforin/granzyme B) production; tumor-derived factors affect miRNAs expression in CD8^+^ T cells, further affect the proliferation/IFN-γ production by CD8^+^ T cells.

Th1 cells are the main CD4^+^ T cell population involved in the response against tumors. The production of IFN-γ is a hallmark of Th1 cells function in the TME. miRNAs such as the miR-17-92 cluster, miR-24, and miR-181 participate in the production of IFN-γ (20, 21) (Figure 1A). The miR-17-92 cluster, for example, plays a key role in controlling Th1 cells responses through multiple coordinated processes. Deficiency of miR-17-92 cluster in CD4^+^ T cells significantly impairs the Th1 cells response to B16 tumor cells, including a decrease in Th1 cells number and IFN-γ production and impairment in the ability of CD4^+^ T cells to help CD8^+^ T cells. Two members of the miR-17-92 cluster, miR-19b and miR-17, cooperate and inhibit tumor progression. Specifically, miR-19b directly targets *PTEN*, enhances the activity of the PI3K/AKT signaling pathway, promotes the proliferation of Th1 cells and the production of IFN-γ, and inhibits the differentiation of iTreg. miR-17 targets *CREB1* and *TGF*β*-II* and inhibits the differentiation of iTreg (20). These data suggest that the inhibition of the miR-17-92 cluster might subvert the immune response against tumors.

miRNAs expressed in tumor cells affect the function of Th1 cells (Figure 1A). For example, miRNAs in tumor-derived microvesicles (MVs)/exosomes such as miR-24-3p, miR-891a, miR-106a-5p, miR-20a-5p, and miR-1908, have been found to impair T cell function by inhibiting Th1 and Th17 differentiation; downregulating the MAPK pathway; affecting the secretion of cytokines such as IL-1β, IL-6, IL-10, IFN-γ, IL-2, and IL-17, and reducing the antitumor effect (22).

Tregs are important in maintaining immunosuppression. Many miRNAs such as miR-21, miR-126, miR-142-3p, miR-146, and miR-155 have been reported to regulate the differentiation, maintenance, and function of Tregs (12, 23–26). Regarding the function of Tregs in the TME, miR-21 has been found to be highly expressed in CCR6^+^ Tregs in tumor tissues from a murine breast cancer model. Silencing of miR-21 altered the enrichment of CCR6^+^ Tregs in the tumor mass and enhanced the antitumor effect of CD8^+^ T cells. Mechanistic evidence has shown that miR-21 targets *PTEN*, alters the activation of the AKT pathway, and reduces the proliferation of CCR6^+^ Tregs, abrogating their immunosuppressive capacity (26). Other miRNAs, such as miR-31 (27), miR-210 (28), and the miR-17-92 cluster (29) have been reported to regulate the expression of Foxp3 and IL-10 and affect the immunosuppressive function of Tregs (Figure 1B), but their roles in the TME remain unclear. This indicates that the targeting of specific miRNAs in Tregs is promising in the development of therapeutic strategies against tumors.

Tumor-derived factors such as miRNAs could also be taken up by Tregs, further affecting the immune response (Figure 1B). The miR-214 secreted by various human cancers and mouse tumor models is delivered to recipient Tregs by MVs, efficiently downregulates *PTEN*, promotes Tregs expansion, and enhances the production of IL-10 *in vitro* (30). Specifically, the authors found that in a lung carcinoma model in nude mice, miR-214 increased the secretion of IL-10 by Tregs and promoted tumor growth. However, when anti-miR-214 antisense oligonucleotides (ASOs) were delivered to mice implanted with tumors, the expansion of Tregs was blocked and tumor growth was inhibited (Figure 1B). This revealed a novel mechanism through which cancer cells actively manipulate the immune response by promoting Tregs expansion (30).

The antitumor effect of CD8^+^ T cells in the TME can be evaluated by the cytokines (mainly IFN-γ) and cytotoxic molecules (mainly perforin and granzyme B) they produce. The process can also be regulated by miRNAs. Several research groups have identified unique miRNAs that regulate CD8^+^ T cell production of IFN-γ, such as miR-29 (31), miR-146a, and miR-155 (32) (Figure 1C). For example, in a mouse melanoma model, researchers found restricted tumor growth in miR-146a-deficient mice and enhanced tumor activity in miR-155-deficient mice. miR-155 seemed to play a more dominant role than that of miR-146a, because in mice lacking both miR-146a and miR-155, CD8^+^ T cells show defects in IFN-γ expression and antitumor immunity, a phenotype similar to that observed in CD8^+^ T cells of miR-155-deficient mice (32). Similarly, another group found that when miR-155 was overexpressed in CD8^+^ T cells, the survival of tumor-challenged mice was prolonged significantly (33).

miRNAs also mediate CD8^+^ T cells effector responses other than IFN-γ production, such as the secretion of perforin and granzyme B (Figure 1C). For example, the miR-17-92 cluster (34) and miR-23a (35) have been reported to regulate the expression of these cytotoxic molecules in CD8^+^ T cells. miR-17-92-deficient CD8^+^ T cells failed to upregulate T-bet and Eomes through an unknown mechanism, which ultimately decreased the production of perforin and granzyme B (34). On the other hand, miR-23a has been found to be upregulated in tumor-infiltrating CD8^+^ T cells of patients with lung cancer, where it acts as a repressor of the transcription factor *BLIMP-1*, which promotes CD8^+^ T cell cytotoxicity. The inhibition of miR-23a enhances granzyme B expression in human CTLs and robustly hinders tumor progression in mice with established melanoma tumors. These data indicate that by modulating different signaling pathways, the miR-17-92 cluster and miR-23a play opposite roles in regulating the function of CD8^+^ T cells, suggesting the multifaceted function of miRNAs in the TME.

Moreover, tumor-derived factors influence the miRNAs expression of CD8^+^ T cells (Figure 1C), tumor-derived TGF-β induce the expression of miR-491 in CD8^+^ T cells. High expression level of miR-491 decreased cell proliferation, increased apoptosis, and decreased IFN-γ production by CD8^+^ T cells. These effects are exerted through the targeting of *Bcl-xL, CDK4*, and *TCF-1*, thereby enhancing tumor immune escape (36).

Immune checkpoint molecules expressed on T cells surfaces such as PD-1, CTLA-4, TIM-3, BTLA, and LAG3 are particularly appealing for cancer therapy. Evidence has shown that the expression of these molecules could be regulated by miRNAs, and they influence tumor progression by different mechanisms (37–42), and representative miRNAs involved in these processes are indicated in Table 1. In addition, checkpoint molecules such as PD-L1 expressed on tumor cells can also be regulated by miRNAs, and they affect the function of immune cells and alter the behavior of tumor cells (43–48), as summarized in Table 2.

**Table 1:** Representative immune cell microRNAs (miRNAs) related to the immune response in the tumor microenvironment (TME).

**miRNAs**	**Host cells**	**Target molecules**	**Expression level**	**Immune-related roles in the TME**	**References**
miR-15a/16	T	PD-1, TIM-3, LAG3	Downregulated	Enhances the activation of tumor-infiltrating CD8^+^T cells	(42)
miR-28	T	PD-1, TIM-3, BTLA	Downregulated	Inhibits T cell exhaustion, regulates the secretion of cytokines (IL-2 and TNF-α)	(39)
miR-138	T	PD-1, CTLA-4	Downregulated	Exerts anti-glioma efficacy	(41)
miR-182	NK	NKG2A NKG2D	Upregulated	Induces NK-cell cytotoxicity (perforin-1 upregulation and increase in cytolytic killing ability)	(38)
miR-1245	NK	NKG2D	Downregulated	Promotes cancer immunosurveillance	(37)
miR-30c	NK	NKG2D CD107a, FasL	Upregulated	Enhances NK cell activation and cytotoxicity to tumor cells	(40)

**Table 2:** Representative tumor cell microRNA (miRNAs) related to immune response in tumor microenvironment (TME).

**miRNAs**	**Host cells**	**Target molecules**	**Expression level**	**Immune-related roles in the TME**	**References**
miR-BART-2, 4, 5, 18, 22	CRC, GC	PD-L1	Upregulated	Suppress immune responses, enhance the secretion of TGF-β and IL-10	(45)
miR-200	NSCLC	PD-L1	Downregulated	Reverses CD8^+^T cell exhaustion	(43)
miR-20b, 21, 130b	CRC	PD-L1	Upregulated	Mitigate T cell activation	(44)
miR-424(322)	OC	PD-L1	Downregulated	Promotes the proliferation of functional cytotoxic CD8^+^ T cells and inhibits MDSCs and Tregs	(47)
miR-34a	AML	PD-L1	Downregulated	Reduces specific T cell apoptosis	(46)
miR-142-5p	PC	PD-L1	Downregulated	Increases CD4^+^T cells and CD8^+^ T cells, decreases PD-1^+^ T cells, increases IFN-γ and TNF-α	(48)

*CRC, colorectal cancer; GC, gastric cancer; NSCLC, non-small cell lung cancer; OC, ovarian cancer; AML, acute myelocytic leukemia; PC, pancreatic cancer*.

Collectively, these data support the notion that the widespread changes in miRNA expression in T cells affect tumorigenesis and determine the behavior of cancer. Therefore, deregulated miRNAs in T cells may be regarded as potential targets in cancer immunotherapy.

#### miRNAs as modulators between NKs and tumor cells

**Figure 2:**
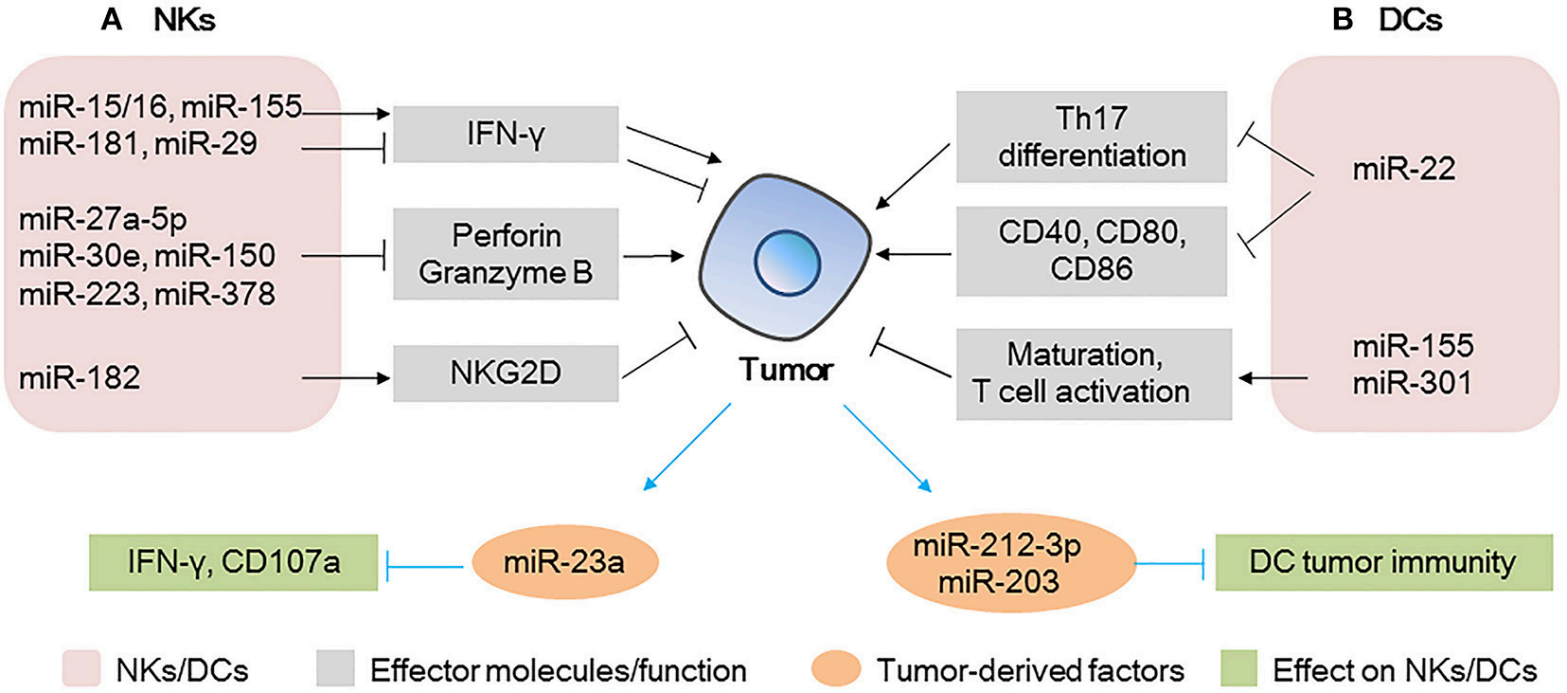
MicroRNAs (miRNAs) act as modulators between natural killer (NKs)/dendritic cells (DCs) and tumor cells **(A)** miRNAs expressed in NKs modulate tumor progression by regulating the production of effector molecules (IFN-γ and perforin/granzyme B) and the activating receptor NKG2D; tumor-derived miRNAs affect the IFN-γ production/CD107 expression in NKs. **(B)** miRNAs expressed in DCs modulate tumor progression by regulating Th17 differentiation, co-stimulatory molecules expression, or T cell activation; tumor-derived miRNAs can be taken up by DCs, affecting the tumor immunity of DCs.

NKs can rapidly respond to the presence of tumor cells and initiate an antitumor immune response by producing effector molecules such as IFN-γ, perforin, and granzyme B (49, 50) (Figure 2A). The most extensively studied individual miRNA in NKs is miR-155, which promotes the production of IFN-γ by targeting *SHIP1* in NKs, increases antitumor activity *in vitro*, and improves the survival of lymphoma-bearing mice *in vivo* (14). Other miRNAs such as miR-15/16, miR-29, and miR-181 also regulate the production of IFN-γ in NKs by different mechanisms (31, 51, 52) (Figure 2A).

A number of groups have reported that miRNAs such as miR-27a-5p, miR-30e, miR-150, miR-223, and miR-378 potentially regulate perforin or granzyme B production or both and impair the antitumor potential of NKs in the TME (53–56). Notably, miR-150-deficient NKs have higher perforin levels and increased NKs cytotoxicity than non-deficient NKs do. Moreover, it has been shown that injection of miR-150-deficient NKs in immune-deficient mice causes a significant reduction in tumor growth and metastasis of B16 melanoma. Thus, the therapeutic control of miR-150 in NKs could enhance NK-based immunotherapy against cancer, providing a better clinical outcome (56).

NKs activation status is also determined by the balance between activating and inhibitory receptors, such as NKG2D and NKG2A, respectively (Figure 2A). Researchers have demonstrated that the NKs of patients with hepatocellular carcinoma (HCC) have higher miR-182 levels than those of healthy subjects. In the same patients, NKG2D and NKG2A were upregulated and downregulated, respectively. Interestingly, miR-182 overexpression in isolated HCC NKs was associated with the upregulation of both receptors, increased production of perforin-1, and cytotoxicity of NKs when these cells were co-cultured with Huh-7 cells. This suggested that, upon overexpression of miR-182, the activation signals of NKG2D override the inhibitory signals of NKG2A, leading to enhanced cytotoxicity of NKs (38). Other miRNAs (40, 57) involved in the regulation of these receptors are summarized in Table 1.

Tumor-derived MVs are key mediators of the interactions between tumor and immune cells (Figure 2A). Berchem et al. (58) found that MVs derived from hypoxic tumor cells are taken up by NKs and affect the immune response. For example, miR-23a in hypoxic tumor-derived MVs operates as an immunosuppressive factor in NKs, by directly targeting *IFN-*γ and *CD107a* in NKs and attenuating NK function. TGF-β secreted by tumor cells can also be transferred to NKs by MVs, decreasing the cell surface expression of NKG2D and inhibiting NK function (58).

Thus, miRNAs expressed in NKs or secreted by tumor cells affect tumor behavior by manipulating the production of effector molecules or the expression of cell surface molecules. These findings highlight the potential role of miRNAs in evading immune surveillance and hence, in tumor progression.

#### miRNAs as modulators between DCs and tumor cells

DCs are central players in the induction of antitumoral immunity, providing critical signals that drive the induction of T cell responses. Notably, miRNAs expressed in DCs, such as miR-22 (59), miR-155 (60), and miR-301 (61) are associated with the antitumor activity of DCs and their immune regulation (Figure 2B). Researchers found that in the B16 mouse melanoma model, the overexpression of miR-22 in DCs leads to faster tumor development, larger tumor size, shorter survival time, and heavier tumors than miR-22 inhibitor group. Specifically, the authors showed that miR-22 decreased the production of IL-6, which in turn, inhibited the polarization of Th17 and the expression of IL-17A, finally promoting tumor growth (59). Furthermore, miR-22 inhibited *p38*, thereby downregulating CD40, CD80, and CD86 and raising the risk of DC dysfunction. Thus, miR-22 has a tumor-promoting effect and impairs the effectiveness of immunotherapy, suggesting that the inhibition of miR-22 could be a strategy to enhance the antitumor activity of DCs. Therefore, miR-22 inhibitors could serve as promising agents to improve the performance of existing DC-based therapeutic tumor vaccines (59).

miR-155, which plays important roles in various cell types, can also regulate the properties of DCs. Researchers have found that miR-155 induction is required for efficient DC maturation and is critical for the ability of DCs to promote antigen-specific T cell activation (60). The enhancement of the activity of miR-155 in ovarian cancer-associated DCs could transform them from immunosuppressive to highly immune stimulatory cells, capable of triggering potent antitumor responses and abrogating the progression of ovarian cancer (62).

miRNAs derived from tumor cells also affect DCs (Figure 2B). miR-212-3p, secreted from pancreatic cancer cells, can be transferred to DCs and inhibit *RFXAP* expression, resulting in decreased expression of MHC-II molecules and induced immune tolerance of DCs (63). Similarly, miR-203, derived from pancreatic cancer-secreted exosomes, can be transferred to DCs, leading to the downregulation of *TLR4* and reducing the production of TNF-α and IL-12, which are necessary for the maturation and differentiation of DCs, respectively, and resulting in weakened cellular immunity (64). These findings suggest that exosome-mediated miRNA transfer between immune and tumor cells plays an important role in the modulation of the TME and that the associated miRNAs might be new potential targets for tumor immunotherapy.

#### miRNAs as modulators between MDSCs and tumor cells

**Figure 3:**
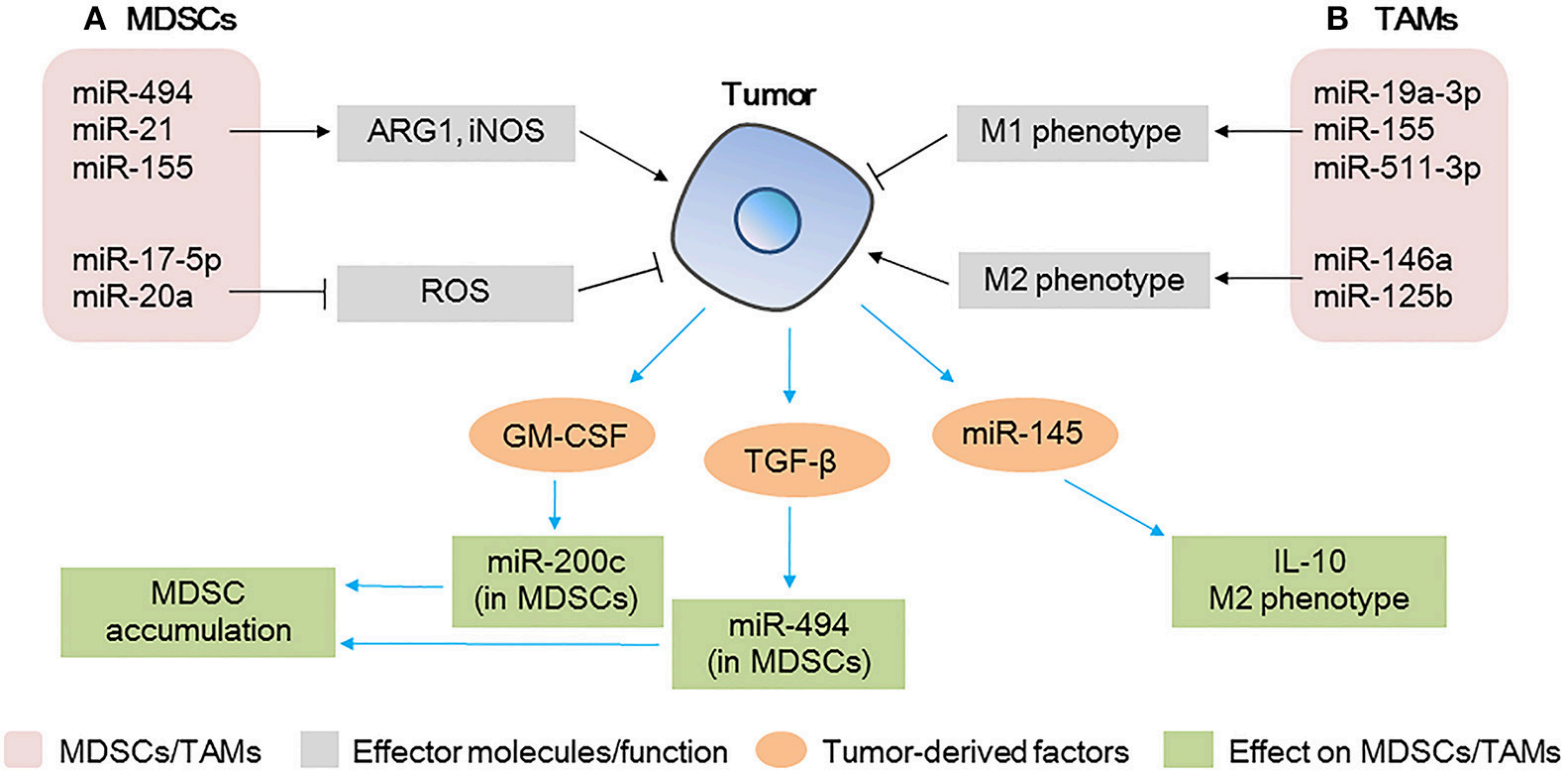
MicroRNAs (miRNAs) act as modulators between myeloid-derived suppressor cells (MDSCs)/tumor-associated macrophages (TAMs) and tumor cells **(A)** miRNAs expressed in MDSCs modulate tumor progression by regulating the expansion/immunosuppressive function of MDSCs; tumor-derived factors affect miRNA expression in MDSCs, affecting the accumulation of MDSCs at the tumor site. **(B)** miRNAs expressed in TAMs modulate tumor progression by regulating the polarization of TAMs; tumor-derived miRNAs affect TAM polarization and IL-10 production.

MDSCs comprise the major cell population that negatively regulates immune responses. MDSCs suppress T cells function through a number of mechanisms involving arginase 1 (ARG1), inducible NO synthase (iNOS), and reactive oxygen species (ROS) (65, 66). It has been demonstrated that some miRNAs such as miR-494, miR-155, and miR-21 are fundamental for the recruitment of MDSCs to the tumor site (Figure 3A), contributing to the modulation of their immunosuppressive function and to tumor growth by regulating the production of ARG1 and iNOS (67, 68). In contrast, miR-17-5p and miR-20a inhibit the expression of STAT3, reduce the production of ROS, and inhibit the immunosuppressive function of MDSCs (69).

Notably, the expression of miR-494 in MDSCs downregulates the protein levels of PTEN, increases the activity of the AKT pathway, and upregulates ARG1 and iNOS, thus contributing to the accumulation of MDSCs in the tumor tissue and promoting tumor cell invasion and metastasis. Contrarily, the knockdown of miR-494 inhibits the activity of MDSCs and inhibits tumor growth and metastasis of 4T1 murine breast cancer *in vivo*, suggesting that miR-494 plays a key role in the expansion and maintenance of tumor-associated MDSCs (67). A different study found that the levels of miR-155 and miR-21 are increased in bone marrow and spleen MDSCs of tumor-bearing mice; the authors showed that the overexpression of miR-155 and miR-21 induced the expansion of both monocytic and granulocytic MDSCs by targeting *SHIP-1* and *PTEN*, increased the production of ARG1 and iNOS, and enhanced immunosuppression (68). These data indicate that miRNAs play critical roles in the events governing the accumulation and function of tumor-expanded MDSCs.

Conversely, tumor-derived factors affect the function of MDSCs (Figure 3A). GM-CSF derived from tumor cells increases miR-200c levels in MDSCs and miR-200c, in turn, promotes the expansion and immunosuppressive activity of MDSCs by targeting *PTEN* and *FOG2*. Specifically, *in vivo* experiments have shown that miR-200c can remarkably promote tumor growth by modifying MDSCs (70). Similarly, TGF-β1, derived from breast cancer cells, increases the levels of miR-494 in MDSCs, enhances CXCR4-mediated MDSC chemotaxis, contributes to the accumulation of MDSCs in tumor tissues, and facilitates the invasion and metastasis of tumor cells (67).

MDSCs are heterogeneous populations of immature and suppressive myeloid cells that expand in nearly all diseases. In the TME, miRNAs regulate the balance between MDSCs and tumor cells and, thus, miRNAs might serve as potential therapeutic targets, both in MDSCs and tumor cells.

#### miRNAs as modulators between TAMs and tumor cells

TAMs are key components of the TME, and they directly affect multiple processes in tumor development. In response to microenvironmental signals, TAMs undergo M1 or M2 polarization and, therefore, exert anti-tumoral or pro-tumoral functions (71). miRNAs are involved in the polarization of TAMs (Figure 3B). The overexpression of miR-155 in TAMs re-programs anti-inflammatory, pro-tumoral M2 TAMs to pro-inflammatory, anti-tumoral M1 macrophages (71). Similarly, in breast cancer TAMs, miR-19a-3p regulates the switch from an M2-like into an M1-like phenotype by targeting *Fra-1, VEGF*, and *STAT3*, contributing to the inhibition of metastasis (72). In contrast, miR-146a promotes the expression of M2 phenotype-associated molecules and promotes 4T1 tumor growth (73). Other miRNAs expressed in TAMs such as miR-142-3p (74), miR-125b (75), and miR-511-3p (76) also play vital roles in tumor progression. These studies show that the modulation of a single miRNA in TAMs could promote the activation of a specific signaling pathway and change the fate of tumor cells in the TME.

Furthermore, a recent study has shown that miRNAs in colorectal cancer cell-derived MVs contribute to tumor development and modulate the TME by regulating the polarization of TAMs (77) (Figure 3B). The authors found that miR-145 secreted by colorectal cancer cells via MVs is taken up by TAMs, targets histone deacetylase 11 (*HDAC11*) and *TLR4*, enhances the production of IL-10, promotes the polarization of TAMs to M2-like macrophages, and promotes tumor progression. In addition, MV-treated macrophages cause significant enlargement of the tumor (77).

The crosstalk of specific miRNAs between different immune and tumor cells has been widely demonstrated in the TME (all the representative miRNAs mentioned in the text related to the immune response in the TME are summarized in Tables 3, 4). Thus, miRNAs are potential targets in immunotherapy, and modulating specific miRNA expression in immune or tumor cells or both may have a significant effect on tumor defense.

**Table 3:** Representative microRNAs (miRNAs) expressed in immune cells related to immune response in tumor microenvironment (TME).

**Tumor-derived/tumor-derived factors induced miRNAs**	**Host cells**	**Immune-related roles in the TME**	**References**
miR-17	Th1 cells	Inhibits the differentiation of iTregs, enhances Th1 response to tumor cells	(20)
miR-19b, 24, 181	Th1 cells	Promote the proliferation of Th1 cells and production of IFN-γ, inhibit the differentiation of iTregs, inhibit tumor progression	(20)
miR-21	Tregs	Reduces the proliferation of Tregs, abrogates immunosuppressive capacity	(26)
miR-31, 210, 17-92 cluster	Tregs	Promote the expression of Foxp3 and IL-10, enhance immunosuppressive function	(27)
			(28)
			(29)
miR-29, 146a, 155	CD8^+^T cells	Promote/inhibit the production of IFN-γ, affect tumor growth	(31)
			(32)
miR-17-92 cluster, 23a	CD8^+^T cells	Promote/inhibit the production of perforin/granzyme B, regulate tumor progression	(34)
			(35)
miR-155 et al	NKs	Promote/inhibit the production of IFN-γ, affect tumor growth	(13)
miR-150 et al	NKs	Inhibit the production of perforin/granzyme B, promote tumor progression	(56)
miR-182	NKs	Promotes the expression of activating receptors NKG2D, enhances cytotoxicity of NKs	(38)
miR-22	DCs	Inhibits polarization of Th17, downregulates CD40, CD80, CD86, decreases the antitumor activity of DCs	(59)
miR-155, 301	DCs	Promote DCs maturation, enhance the capacity of T cell activation, abrogate tumor progression	(60) (62) (61)
miR-494, 21, 155	MDSCs	Upregulate ARG1 and iNOS, enhance immunosuppression	(67) (68)
miR-17-5p, 20a	MDSCs	Reduce the production of ROS, inhibit the immunosuppressive function of MDSCs	(69)
miR-146a, 155 et al	TAMs	Regulate the polarization of TAMs to M1/M2 phenotype, change the fate of tumor cells	(71) (73)

**Table 4:** Representative tumor-derived/tumor-derived factors induced microRNAs (miRNAs) related to immune response in tumor microenvironment (TME).

**Tumor-derived/tumor -derived factors induced miRNAs**	**Host cells**	**Immune-related roles in the TME**	**References**
miR-24-3p	Th1 cells	Affects the secretion of cytokines such as IFN-γ, reduces the antitumor effect	(22)
miR-214	Tregs	Increases the secretion of IL-10 by Tregs, promotes tumor growth	(30)
miR-491	CD8^+^ T cells	Decreases proliferation, IFN-γ production, increases apoptosis of CD8^+^ T cells, enhances tumor immune escape	(36)
miR-23a	NKs	Reduces IFN-γ production and CD107a expression of NKs, enhances immunosuppressive effect	(58)
miR-212-3p, 203	DCs	Decrease expression of MHC-II, inhibit maturation and differentiation, induce immune tolerance of DCs	(63) (64)
miR-200c, 494	MDSCs	Promote MDSCs expansion and accumulation of MDSCs in tumor tissues, facilitate tumor invasion and metastasis	(67) (70)
miR-145	TAMs	Enhances the production of IL-10 and the polarization of TAMs to M2-like macrophages, promotes tumor progression	(77)

### miRNAs as modulators between CAEs and tumor cells

**Figure 4:**
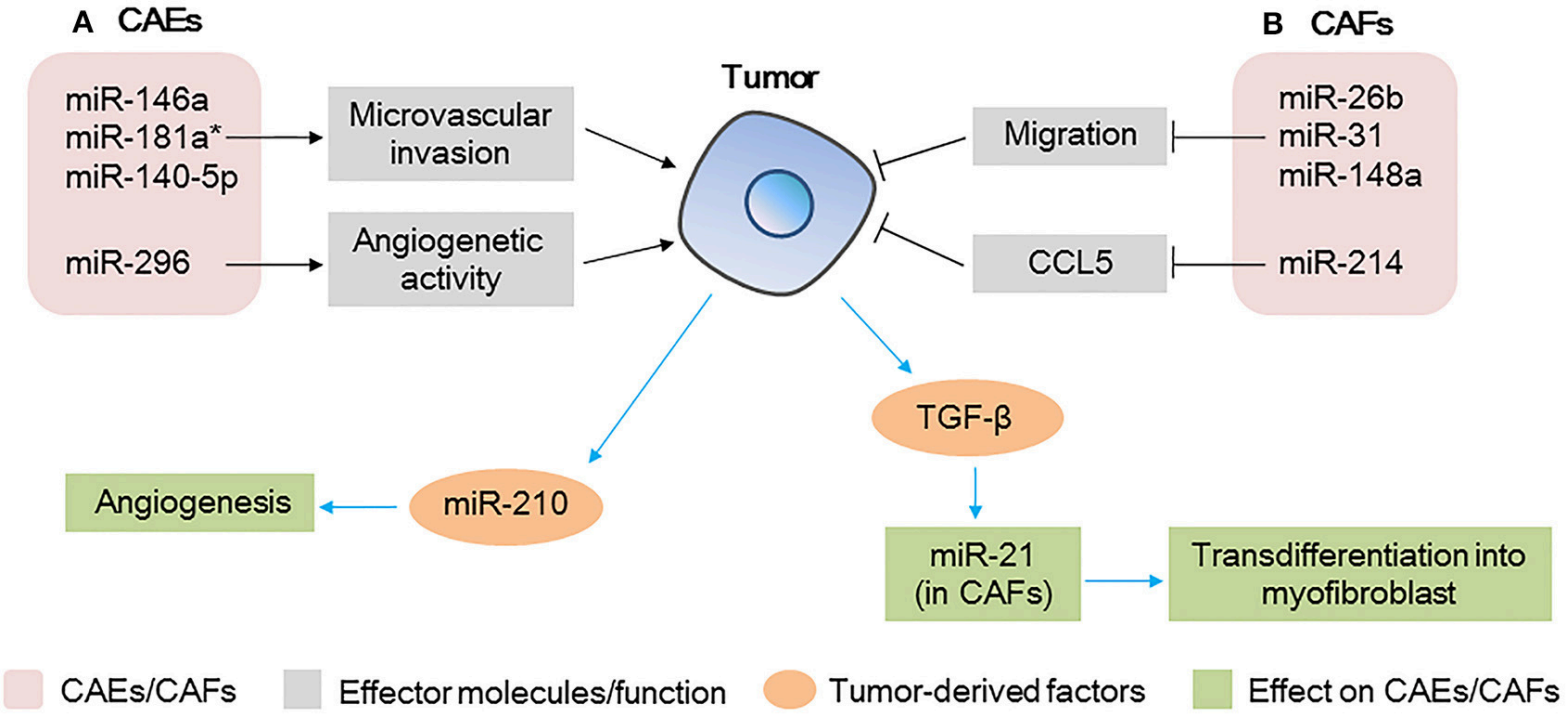
microRNAs (miRNAs) act as modulators between cancer-associated endothelial cells (CAEs)/cancer-associated fibroblasts (CAFs) and tumor cells **(A)** miRNAs expressed in CAEs modulate tumor progression by regulating the microvascular invasion and angiogenesis activity of CAEs; tumor-derived miRNAs affect the angiogenesis capacity of CAEs. **(B)** miRNAs expressed in CAFs modulate tumor progression by regulating the migration and chemokine production; tumor-derived factors affect miRNAs expression in CAFs, further affect the transdifferentiation of myofibroblasts.

Endothelial cells are important components of tumor stroma. They line the interior surface of blood vessels and lymphatic vessels and in TME, the unregulated growth and migration of CAEs contribute considerably to angiogenesis and regulation of tumor metastasis (78). Altered expression of miRNAs in CAEs also affect tumor progression as shown in Figure 4A. In a HCC model, when endothelial cells were co-cultured with HCC cells, miR-146a, miR-181a^*^, and miR-140-5p were significantly upregulated and miR-302c was downregulated. Among these miRNAs, the upregulation of miR-146a was found to promote CAEs proliferation, vascularization, and tumor growth (79). Mechanism proof studies revealed that miR-146a promoted the expression of platelet-derived growth factor receptor α (PDGFRA) in CAEs, which was mediated by BRCA1 (79). Moreover, overexpression of PDGFRA in the CAEs of HCC tissues was found to be associated with microvascular invasion and predicted a poorer prognosis, indicating that miR-146a plays a key role in regulating the angiogenetic activity of CAEs in HCC (79). Other miRNAs such as miR-296 were also found to be upregulated in CAEs in a glioma model, which was found to promote angiogenesis by increasing endothelial cells and finally promote tumor growth (80).

miRNAs derived from tumor cells could also affect angiogenesis of CAEs as shown in Figure 4A. In a study of patients with HCC, a high level of miR-210-3p (miR-210) was detected in the exosomes isolated from the sera of the patients. Further studies demonstrated that HCC cell-derived exosomal miR-210 could be delivered to CAEs, enhancing angiogenesis (81). Subsequent *in vivo* studies revealed that subcutaneous tumor xenografts treated with HCC cells-derived exosomal miR-210 showed much more vessels and a larger tumor volume than control group without exosomal miRNA (81). These findings revealed that miR-210 may be used as a potential therapeutic target in anti-HCC therapy.

Endothelial cells are critical for angiogenesis and regulating tumor metastasis, deregulated miRNAs expression in CAEs do affect the tumor progression, this reminds us that targeting miRNAs in CAEs could be a new approach for cancer therapy.

### miRNAs as modulators between CAFs and tumor cells

In the TME, fibroblasts regulate angiogenesis and metastasis of tumor cells, and miRNAs are notable key regulators of the tumor promoting function of CAFs as shown in Figure 4B. A miRNA array analysis of CAFs in breast cancer showed that six miRNAs were significantly downregulated, consisting of miR-7f, miR-7g, miR-15b, miR-26b, miR-30b, and miR-107. Furthermore, among these miRNAs, miR-26b was the most highly deregulated, and its downregulation in CAFs promoted the migration of fibroblasts and further promoted the invasion of breast cancer cells (82). Similarly, miR-31 and miR148a were also found to be downregulated in CAFs in endometrial cancer, which finally promoted tumor invasiveness and growth (83, 84). Moreover, miR-214 in CAFs was reported to be downregulated in patients with ovarian cancer and in addition, the expression of the pro-tumorgenetic chemokine CCL5 was enhanced, finally promoting the invasion and growth of ovarian cancer cells (85).

Tumor-derived factors such as TGF-β could also regulate the expression of miRNAs in CAFs, further affecting tumor progression as shown in Figure 4B. For example, miR-21 was reported to be upregulated in CAFs by colorectal tumor-derived TGF-β, increased expression of miR-21 was shown to upregulate α-SMA, which promotes CAFs transdifferentiation into myofibroblasts, resulting in increased proliferative capacity of colorectal cancer (86). TGF-β was also shown to upregulate CAFs miR-143 in gastric cancer, which finally promoted tumorigenesis by enhancing the expression of collagen type III (87).

Taken together, these data highlight the importance of miRNAs deregulation in CAEs and CAFs, and identify a potential application for stromal miRNAs as biomarkers in cancer.

## A perspective on miRNAs in cancer therapy

Recent and ongoing investigations have enhanced our knowledge of the function of miRNAs in tumor biology and immune cell development. miRNAs can affect tumor progression by regulating gene expression in tumor cells or modulating the function of immune cells in the TME. Because of their multiple functions in physiological processes, miRNAs are considered as potential biomarkers in diagnostics and prognosis of various cancers (7). In a study of 391 patients with non-small cell lung cancer (NSCLC), serum miRNAs were shown to serve as predictors and biomarkers of survival for patients with advanced NSCLC (88).

miRNAs are also proposed to be therapeutic targets. miRNAs that act as suppressors of tumorigenesis need to be overexpressed, while those inducing tumorigenesis need to be silenced. The current strategy to overexpress or inhibit miRNAs uses the delivery of synthetic oligoribonucleotides, which mimic the native miRNA duplex or are antisense RNA. Different types of vehicles have been synthesized as carriers of miRNA mimics and antagonists, including liposomes, polymers, nanoparticles, and viral agents (89); they have been found to improve the efficiency and specificity of the systemic delivery of miRNA (90–92). We have indicated that several examples showing tumor-derived MVs could modulate the immune response. Thus, cell-derived exosomes containing immune-related miRNAs might be used as therapeutic agents to enhance antitumor immunity.

While many studies indicate the possible clinical application of miRNAs (93), clinical trials have tested the efficacy of miRNA-based cancer therapeutics. MRX34, a synthetic miR-34a, was used in a phase I clinical trial in patients with primary liver cancer and liver metastasis in 2013. In the study, miR-34a, a tumor suppressor downstream of p53, was overexpressed by loading it into liposomal nanoparticles (94). Although it originally seemed promising, the study was halted in 2016 because of multiple adverse immune-related events. In addition, many ASOs have also been tested in clinical trials. These therapies are RNA-based (not miRNA-based); they do not knockdown or overexpress miRNAs but target oncogenes such as *Bcl-2* (NCT00285103) in CLL, *STAT3* (NCT01563302) in DLBCL and lymphoma, and *MYC* (NCT02314052) in HCC. These therapeutics have already reached phase I/II (https://clinicaltrials.gov/).

Although miRNA-based therapies provide hope for their clinical application, some issues need to be considered: (1) miRNAs target molecules in both normal and cancer cells, and their efficacy and safety need to be tested; (2) miRNA-based agents are easily degraded and, therefore, new approaches are necessary to enhance their stability and prevent degradation by nucleases; (3) because the therapeutics need to reach target tissues, their half-life has to be long enough; and (4) to avoid miRNA-based therapeutics being taken up by normal cells, proper delivery systems need to be developed.

## Conclusion

miRNAs are multifunctional molecules that play essential roles in physiological processes, and the deregulation of miRNAs is often associated with human diseases. In this review, we have discussed the crosstalk of miRNAs between immune cells, CAEs, CAFs, and tumor cells in the TME and have summarized the potential clinical application of miRNA-based therapeutics. While most of the investigations focus on the deregulation of miRNAs in cancer cells, the altered expression of miRNAs in immune cells, CAEs and CAFs in the TME can also affect tumor progression. Thus, the identification of these altered miRNAs may lead to the discovery of novel biomarkers for immunotherapy. Overall, in consideration of the complex functions of miRNAs in different cell types, we believe that in the future, miRNA-based therapeutics will be used to treat a broad range of human diseases.

## Author contributions

NY carried out the primary literature search, wrote and revised the manuscript, and created the illustrations. SZ, XPL, YQ, and Y-JL were involved in the preparation and revision of the manuscript. JTC initiated the concept and supervised the writing and revision of the manuscript. All authors read and approved the final manuscript.

### Conflict of interest statement

The authors declare that the research was conducted in the absence of any commercial or financial relationships that could be construed as a potential conflict of interest.

## References

[1] Bartel DP. MicroRNAs: genomics, biogenesis, mechanism, and function. Cell (2004) 116:281–97. 10.1016/S0092-8674(04)00045-5 14744438

[2] Lee RC Feinbaum RL Ambros V. The *C. elegans* heterochronic gene lin-4 encodes small RNAs with antisense complementarity to lin-14. Cell (1993). 75:843–54. 8252621 10.1016/0092-8674(93)90529-y

[3] Hammond SM. An overview of microRNAs. Adv Drug Deliv Rev. (2015) 87:3–14. 10.1016/j.addr.2015.05.001 25979468 PMC4504744

[4] Lundstrom K Micro-RNA in disease and gene therapy. Curr Drug Discov Technol. (2011) 82:76–86. 10.2174/157016311795563857 21513487

[5] Friedman RC Farh KKH Burge CB Bartel DP. Most mammalian mRNAs are conserved targets of microRNAs. Genome Res. (2008) 19:92–105. 10.1101/gr.082701.108 18955434 PMC2612969

[6] Tüfekci KU Öner MG Meuwissen RLJ Genç S. The role of MicroRNAs in human Diseases. Methods Mol Biol. (2014) 1107:33–50. 10.1007/978-1-62703-748-83 24272430

[7] Di Leva G Garofalo M Croce CM. MicroRNAs in cancer. Annu Rev Pathol. (2014) 9:287–314. 10.1146/annurev-pathol-012513-104715 24079833 PMC4009396

[8] Calin GA Dumitru CD Shimizu M Bichi R Zupo S Noch E. Frequent deletions and down-regulation of micro- RNA genes miR15 and miR16 at 13q14 in chronic lymphocytic leukemia. Proc Natl Acad Sci USA (2002) 99:15524–9. 10.1073/pnas.242606799 12434020 PMC137750

[9] Lawrie CH Gal S Dunlop HM Pushkaran B Liggins AP Pulford K. Detection of elevated levels of tumour-associated microRNAs in serum of patients with diffuse large B-cell lymphoma. Br J Haematol. (2008) 141:672–5. 10.1111/j.1365-2141.2008.07077.x 18318758

[10] Adams BD Parsons C Walker L Zhang WC Slack FJ. Targeting noncoding RNAs in disease. J Clin Invest. (2017) 127:761–71. 10.1172/JCI84424 28248199 PMC5330746

[11] Rodriguez A Vigorito E Clare S Warren MV Couttet P Soond DR. Requirement of bic/microRNA-155 for normal immune function. Science (2007) 316:608–11. 10.1126/science.1139253 17463290 PMC2610435

[12] Lu LF Thai TH Calado DP Chaudhry A Kubo M Tanaka K. Foxp3-dependent microRNA155 confers competitive fitness to regulatory T cells by targeting SOCS1 protein. Immunity (2009) 30:80–91. 10.1016/j.immuni.2008.11.010 19144316 PMC2654249

[13] Trotta R Chen L Ciarlariello D Josyula S Mao C Costinean S. miR-155 regulates IFN-gamma production in natural killer cells. Blood (2012) 119:3478–85. 10.1182/blood-2011-12-398099 22378844 PMC3325038

[14] Trotta R Chen L Costinean S Josyula S Mundy-Bosse BL Ciarlariello D. Overexpression of miR-155 causes expansion, arrest in terminal differentiation and functional activation of mouse natural killer cells. Blood (2013) 121:3126–34. 10.1182/blood-2012-12-467597 23422749 PMC3630828

[15] Wang J Iwanowycz S Yu F Jia X Leng S Wang Y. microRNA-155 deficiency impairs dendritic cell function in breast cancer. Oncoimmunology (2016) 5:e1232223. 10.1080/2162402X.2016.1232223 27999745 PMC5139631

[16] Chen S Zhang Y Kuzel TM Zhang B. Regulating tumor myeloid-derived suppressor cells by microRNAs. Cancer Cell Microenviron. (2015) 2:e637. 10.14800/ccm.637 26005707 PMC4440580

[17] He M Xu Z Ding T Kuang DM Zheng L. MicroRNA-155 regulates inflammatory cytokine production in tumor-associated macrophages via targeting C/EBPbeta. Cell Mol Immunol. (2009) 6:343–35. 10.1038/cmi.2009.45 19887047 PMC4003217

[18] Baltimore D Boldin MP O'Connell RM Rao DS Taganov KD. MicroRNAs: new regulators of immune cell development and function. Nat Immunol. (2008) 9:839–45. 10.1038/ni.f.209 18645592

[19] Baumjohann D Ansel KM MicroRNA-mediated regulation of T helper cell differentiation and plasticity. Nat Rev Immunol. (2013) 139:666–78. 10.1038/nri3494 PMC3980848 23907446

[20] Jiang S Li C Olive V Lykken E Feng F Sevilla J. Molecular dissection of the miR-17–92 cluster's critical dual roles in promoting Th1 responses and preventing inducible Treg differentiation. Blood (2011) 118:5487–97. 10.1182/blood-2011-05-355644 21972292 PMC3217351

[21] Fayyad-Kazan H Hamade E Rouas R Najar M Fayyad-Kazan M El Zein N. Downregulation of microRNA-24 and−181 parallels the upregulation of IFN-gamma secreted by activated human CD4 lymphocytes. Hum Immunol. (2014) 75:677–85. 10.1016/j.humimm.2014.01.007 24704866

[22] Ye SB Li ZL Luo DH Huang BJ Chen YS Zhang XS. Tumor-derived exosomes promote tumor progression and T-cell dysfunction through the regulation of enriched exosomal microRNAs in human nasopharyngeal carcinoma. Oncotarget (2014) 5:5439–52. 10.18632/oncotarget.2118 24978137 PMC4170615

[23] Huang B Zhao J Lei Z Shen S Li D Shen GX. miR-142–3p restricts cAMP production in CD4+CD25- T cells and CD4+CD25+ TREG cells by targeting AC9 mRNA. EMBO Rep. (2009) 10:180–5. 10.1038/embor.2008.224 19098714 PMC2637310

[24] Lu LF Boldin MP Chaudhry A Lin LL Taganov KD Hanada T. Function of miR-146a in controlling treg cell-mediated regulation of Th1 responses. Cell (2010) 142:914–29. 10.1016/j.cell.2010.08.012 20850013 PMC3049116

[25] Qin A Wen Z Zhou Y Li Y Li Y Luo J. MicroRNA-126 regulates the induction and function of CD4(+) Foxp3(+) regulatory T cells through PI3K/AKT pathway. J Cell Mol Med. (2013) 17:252–64. 10.1111/jcmm.12003 23301798 PMC3822588

[26] Hu Y Wang C Li Y Zhao J Chen C Zhou Y. MiR-21 controls *in situ* expansion of CCR6(+) regulatory T cells through PTEN/AKT pathway in breast cancer. Immunol Cell Biol. (2015) 93:753–64. 10.1038/icb.2015.37 25735723

[27] Rouas R Fayyad-Kazan H El Zein N Lewalle P Rothe F Simion A Human natural Treg microRNA signature: role of microRNA-31 and microRNA-21 in FOXP3 expression. Eur J Immunol. (2009) 396:1608–18. 10.1002/eji.200838509 19408243

[28] Zhao M Wang LT Liang GP Zhang P Deng XJ Tang Q. Up-regulation of microRNA-210 induces immune dysfunction via targeting FOXP3 in CD4(+) T cells of psoriasis vulgaris. Clin Immunol. (2014) 150:22–30. 10.1016/j.clim.2013.10.009 24316592

[29] de Kouchkovsky D Esensten JH Rosenthal WL Morar MM Bluestone JA Jeker LT microRNA-17–92 regulates IL-10 production by regulatory T cells and control of experimental autoimmune encephalomyelitis. J Immunol. (2013) 191:1594–605. 10.4049/jimmunol.1203567 23858035 PMC4160833

[30] Yin Y Cai X Chen X Liang H Zhang Y Li J. Tumor-secreted miR-214 induces regulatory T cells: a major link between immune evasion and tumor growth. Cell Res. (2014) 24:1164–80. 10.1038/cr.2014.121 25223704 PMC4185347

[31] Ma F Xu S Liu X Zhang Q Xu X Liu M. The microRNA miR-29 controls innate and adaptive immune responses to intracellular bacterial infection by targeting interferon-gamma. Nat Immunol. (2011) 12:861–9. 10.1038/ni.2073 21785411

[32] Huffaker TB Hu R Runtsch MC Bake E Chen X Zhao J. Epistasis between microRNAs 155 and 146a during T cell-mediated antitumor immunity. Cell Rep. (2012) 2:1697–709. 10.1016/j.celrep.2012.10.025 23200854 PMC3628775

[33] Dudda JC Salaun B Ji Y Palmer DC Monnot GC Merck E. MicroRNA-155 is required for effector CD8+ T cell responses to virus infection and cancer. Immunity (2013) 38:742–53. 10.1016/j.immuni.2012.12.006 23601686 PMC3788592

[34] Khan AA Penny LA Yuzefpolskiy Y Sarkar S Kalia V. MicroRNA-17~92 regulates effector and memory CD8 T-cell fates by modulating proliferation in response to infections. Blood (2013) 121:4473–83. 10.1182/blood-2012-06-435412 23596046

[35] Lin R Chen L Chen G Hu C Jiang S Sevilla J. Targeting miR-23a in CD8+ cytotoxic T lymphocytes prevents tumor-dependent immunosuppression. J Clin Invest. (2014) 124:5352–67. 10.1172/JCI76561 25347474 PMC4348954

[36] Yu T Zuo QF Gong L Wang LN Zou QM Xiao B. MicroRNA-491 regulates the proliferation and apoptosis of CD8(+) T cells. Sci Rep. (2016) 6:30923. 10.1038/srep30923 27484289 PMC4971478

[37] Espinoza JL Takami A Yoshioka K Nakata K Sato T Kasahara Y. Human microRNA-1245 down-regulates the NKG2D receptor in natural killer cells and impairs NKG2D-mediated functions. Haematologica (2012) 97:1295–303. 10.3324/haematol.2011.058529 22491735 PMC3436229

[38] Abdelrahman MM Fawzy IO Bassiouni AA Gomaa AI Esmat G Waked I. Enhancing NK cell cytotoxicity by miR-182 in hepatocellular carcinoma. Hum Immunol. (2016) 77:667–73. 10.1016/j.humimm.2016.04.020 27262453

[39] Li Q Johnston N Zheng X Wang H Zhang X Gao D. miR-28 modulates exhaustive differentiation of T cells through silencing programmed cell death-1 and regulating cytokine secretion. Oncotarget (2016) 7:53735–50. 10.18632/oncotarget.10731 27447564 PMC5288217

[40] Ma Y Gong J Liu Y Guo W Jin B Wang X. MicroRNA-30c promotes natural killer cell cytotoxicity via up-regulating the expression level of NKG2D. Life Sci. (2016) 151:174–81. 10.1016/j.lfs.2016.03.012 26968781

[41] Wei J Nduom EK Kong LY Hashimoto Y Xu S Gabrusiewicz K. MiR-138 exerts anti-glioma efficacy by targeting immune checkpoints. Neuro Oncol. (2016) 18:639–48. 10.1093/neuonc/nov292 26658052 PMC4827047

[42] Yang J Liu R Deng Y Qian J Lu Z Wang Y. MiR-15a/16 deficiency enhances anti-tumor immunity of glioma-infiltrating CD8+ T cells through targeting mTOR. Int J Cancer (2017) 141:2082–92. 10.1002/ijc.30912 28758198

[43] Chen L Gibbons DL Goswami S Cortez MA Ahn Y-H Byers LA. Metastasis is regulated via microRNA-200/ZEB1 axis control of tumour cell PD-L1 expression and intratumoral immunosuppression. Nat Commun. (2014) 5:5241. 10.1038/ncomms6241 25348003 PMC4212319

[44] Zhu J Chen L Zou L Yang P Wu R Mao Y. MiR-20b,−21, and−130b inhibit PTEN expression resulting in B7-H1 over-expression in advanced colorectal cancer. Hum Immunol. (2014) 75:348–53. 10.1016/j.humimm.2014.01.006 24468585

[45] Pandya D Mariani M He S Andreoli M Spennato M Dowell-Martino C. Epstein-barr virus microRNA expression increases aggressiveness of solid malignancies. PLoS ONE (2015) 10:e0136058. 10.1371/journal.pone.0136058 26375401 PMC4573609

[46] Wang X Li J Dong K Lin F Long M Ouyang Y. Tumor suppressor miR-34a targets PD-L1 and functions as a potential immunotherapeutic target in acute myeloid leukemia. Cell Signal (2015) 27:443–52. 10.1016/j.cellsig.2014.12.003 25499621

[47] Xu S Tao Z Hai B Liang H Shi Y Wang T. miR-424(322) reverses chemoresistance via T-cell immune response activation by blocking the PD-L1 immune checkpoint. Nat Commun. (2016) 7:11406. 10.1038/ncomms11406 27147225 PMC4858750

[48] Jia L Xi Q Wang H Zhang Z Liu H Cheng Y. miR-142–5p regulates tumor cell PD-L1 expression and enhances anti-tumor immunity. Biochem Biophys Res Commun. (2017) 488:425–31. 10.1016/j.bbrc.2017.05.074 28511795

[49] Smyth MJ Hayakawa Y Takeda K Yagita H. New aspects of natural-killer-cell surveillance and therapy of cancer. Nat Rev Cancer (2002) 2:850–61. 10.1038/nrc928 12415255

[50] Ni F Guo C Sun R Fu B Yang Y Wu L. MicroRNA transcriptomes of distinct human NK cell populations identify miR-362–5p as an essential regulator of NK cell function. Sci Rep. (2015) 5:9993. 10.1038/srep09993 25909817 PMC4408982

[51] Cichocki F Felices M McCullar V Presnell SR Al-Attar A Lutz CT. Cutting edge: microRNA-181 promotes human NK cell development by regulating Notch signaling. J Immunol. (2011) 187:6171–5. 10.4049/jimmunol.1100835 22084432 PMC3237765

[52] Sullivan RP Leong JW Schneider SE Ireland AR Berrien-Elliott MM Singh A. MicroRNA-15/16 antagonizes Myb to control NK cell maturation. J Immunol. (2015) 195:2806–17. 10.4049/jimmunol.1500949 26268657 PMC4561212

[53] Fehniger TA Wylie T Germino E Leong JW Magrini VJ Koul S. Next-generation sequencing identifies the natural killer cell microRNA transcriptome. Genome Res. (2010) 20:1590–604. 10.1101/gr.107995.110 20935160 PMC2963822

[54] Kim TD Lee SU Yun S Sun HN Lee SH Kim JW. Human microRNA-27a^*^ targets Prf1 and GzmB expression to regulate NK-cell cytotoxicity. Blood (2011) 118:5476–86. 10.1182/blood-2011-04-347526 21960590 PMC3217350

[55] Wang P Gu Y Zhang Q Han Y Hou J Lin L. Identification of resting and type I IFN-activated human NK cell miRNomes reveals microRNA-378 and microRNA-30e as negative regulators of NK cell cytotoxicity. J Immunol. (2012) 189:211–21. 10.4049/jimmunol.1200609 22649192

[56] Kim N Kim M Yun S Doh J Greenberg PD Kim TD. MicroRNA-150 regulates the cytotoxicity of natural killers by targeting perforin-1. J Allergy Clin Immunol. (2014) 134:195–203. 10.1016/j.jaci.2014.02.018 24698324 PMC4125537

[57] Espinoza JL Nguyen VH Ichimura H Pham TT Nguyen CH Pham TV. A functional polymorphism in the NKG2D gene modulates NK-cell cytotoxicity and is associated with susceptibility to human papilloma virus-related cancers. Sci Rep. (2016) 6:39231. 10.1038/srep39231 27995954 PMC5172372

[58] Berchem G Noman MZ Bosseler M Paggetti J Baconnais S Le Cam E. Hypoxic tumor-derived microvesicles negatively regulate NK cell function by a mechanism involving TGF-beta and miR23a transfer. Oncoimmunology (2016) 5:e1062968. 10.1080/2162402X.2015.1062968 27141372 PMC4839360

[59] Liang X Liu Y Mei S Zhang M Xin J Zhang Y. MicroRNA-22 impairs anti-tumor ability of dendritic cells by targeting p38. PLoS ONE (2015) 10:e0121510. 10.1371/journal.pone.0121510 25826372 PMC4380340

[60] Dunand-Sauthier I Santiago-Raber ML Capponi L Vejnar CE Schaad O Irla M. Silencing of c-Fos expression by microRNA-155 is critical for dendritic cell maturation and function. Blood (2011) 117:4490–500. 10.1182/blood-2010-09-308064 21385848

[61] Pyfferoen L Mestdagh P Vergote K De Cabooter N Vandesompele J Lambrecht BN. Lung tumours reprogram pulmonary dendritic cell immunogenicity at the microRNA level. Int J Cancer (2014) 135:2868–77. 10.1002/ijc.28945 24789737

[62] Cubillos-Ruiz JR Baird JR Tesone AJ Rutkowski MR Scarlett UK Camposeco-Jacobs AL. Reprogramming tumor-associated dendritic cells *in vivo* using miRNA mimetics triggers protective immunity against ovarian cancer. Cancer Res. (2012) 72:1683–93. 10.1158/0008-5472.CAN-11-3160 22307839 PMC3319850

[63] Ding G Zhou L Qian Y Fu M Chen J Chen J. Pancreatic cancer-derived exosomes transfer miRNAs to dendritic cells and inhibit RFXAP expression via miR-212–3p. Oncotarget (2015) 6:29877–88. 10.18632/oncotarget.4924 26337469 PMC4745769

[64] Zhou M Chen J Zhou L Chen W Ding G Cao L. Pancreatic cancer derived exosomes regulate the expression of TLR4 in dendritic cells via miR-203. Cell Immunol. (2014) 292:65–9. 10.1016/j.cellimm.2014.09.004 25290620

[65] Bronte V Zanovello P. Regulation of immune responses by L-arginine metabolism. Nat Rev Immunol. (2005) 5:641–54. 10.1038/nri1668 16056256

[66] Gabrilovich DI Nagaraj S. Myeloid-derived suppressor cells as regulators of the immune system. Nat Rev Immunol. (2009) 9:162–74. 10.1038/nri2506 19197294 PMC2828349

[67] Liu Y Lai L Chen Q Song Y Xu S Ma F. MicroRNA-494 is required for the accumulation and functions of tumor-expanded myeloid-derived suppressor cells via targeting of PTEN. J Immunol. (2012) 188:5500–10. 10.4049/jimmunol.1103505 22544933

[68] Li L Zhang J Diao W Wang D Wei Y Zhang CY. MicroRNA-155 and MicroRNA-21 promote the expansion of functional myeloid-derived suppressor cells. J Immunol. (2014) 192:1034–43. 10.4049/jimmunol.1301309 24391219

[69] Zhang M Liu Q Mi S Liang X Zhang Z Su X. Both miR-17–5p and miR-20a alleviate suppressive potential of myeloid-derived suppressor cells by modulating STAT3 expression. J Immunol. (2011) 186:4716–24. 10.4049/jimmunol.1002989 21383238

[70] Mei S Xin J Liu Y Zhang Y Liang X Su X. MicroRNA-200c promotes suppressive potential of myeloid-derived suppressor cells by modulating PTEN and FOG2 expression. PLoS ONE (2015) 10:e0135867. 10.1371/journal.pone.0135867 26285119 PMC4540422

[71] Cai X Yin Y Li N Zhu D Zhang J Zhang CY. Re-polarization of tumor-associated macrophages to pro-inflammatory M1 macrophages by microRNA-155. J Mol Cell Biol. (2012) 4:341–3. 10.1093/jmcb/mjs044 22831835

[72] Yang J Zhang Z Chen C Liu Y Si Q Chuang TH. MicroRNA-19a-3p inhibits breast cancer progression and metastasis by inducing macrophage polarization through downregulated expression of Fra-1 proto-oncogene. Oncogene (2014) 33:3014–23. 10.1038/onc.2013.258 23831570

[73] Li Y Zhao L Shi B Ma S Xu Z Ge Y. Functions of miR-146a and miR-222 in tumor-associated macrophages in breast cancer. Sci Rep. (2015) 5:18648. 10.1038/srep18648 26689540 PMC4686897

[74] Sonda N Simonato F Peranzoni E Cali B Bortoluzzi S Bisognin A miR-142–3p prevents macrophage differentiation during cancer-induced myelopoiesis. Immunity (2013) 386:1236–49. 10.1016/j.immuni.2013.06.004 23809164

[75] Chaudhuri AA So AY-L Sinha N Gibson WSJ Taganov KD O'Connell RM. MicroRNA-125b potentiates macrophage activation. J Immunol. (2011) 187:5062–8. 10.4049/jimmunol.1102001 22003200 PMC3208133

[76] Squadrito ML Pucci F Magri L Moi D Gilfillan GD Ranghetti A. miR-511–3p modulates genetic programs of tumor-associated macrophages. Cell Rep. (2012) 1:141–54. 10.1016/j.celrep.2011.12.005 22832163

[77] Shinohara H Kuranaga Y Kumazaki M Sugito N Yoshikawa Y Takai T. Regulated Polarization of tumor-associated macrophages by miR-145 via colorectal cancer-derived extracellular vesicles. J Immunol. (2017) 199:1505–15. 10.4049/jimmunol.1700167 28696255

[78] Junttila MR de Sauvage FJ. Influence of tumour micro-environment heterogeneity on therapeutic response. Nature (2013) 501:346–54. 10.1038/nature12626 24048067

[79] Zhu K Pan Q Zhang X Kong LQ Fan J Dai Z. MiR-146a enhances angiogenic activity of endothelial cells in hepatocellular carcinoma by promoting PDGFRA expression. Carcinogenesis (2013) 34:2071–9. 10.1093/carcin/bgt160 23671131

[80] Plummer PN Freeman R Taft RJ Vider J Sax M Umer BA. MicroRNAs regulate tumor angiogenesis modulated by endothelial progenitor cells. Cancer Res. (2013) 73:341–52. 10.1158/0008-5472.CAN-12-0271 22836757

[81] Lin XJ Fang JH Yang XJ Zhang C Yuan Y Zheng L. Hepatocellular carcinoma cell-secreted exosomal MicroRNA-210 promotes angiogenesis *in vitro* and *in vivo*. Mol Ther Nucleic Acids (2018) 11:243–52. 10.1016/j.omtn.2018.02.014 29858059 PMC5992447

[82] Verghese ET Drury R Green CA Holliday DL Lu X Nash C. MiR-26b is down-regulated in carcinoma-associated fibroblasts from ER-positive breast cancers leading to enhanced cell migration and invasion. J Pathol. (2013) 231:388–99. 10.1002/path.4248 23939832 PMC4030585

[83] Aprelikova O Yu X Palla J Wei BR John S Yi M. The role of miR-31 and its target gene SATB2 in cancer-associated fibroblasts. Cell Cycle (2010) 9:4387–98. 10.4161/cc.9.21.13674 20980827 PMC3055190

[84] Aprelikova O Palla J Hibler B Yu X Greer YE Yi M. Silencing of miR-148a in cancer-associated fibroblasts results in WNT10B-mediated stimulation of tumor cell motility. Oncogene (2013) 32:3246–53. 10.1038/onc.2012.351 22890324 PMC3711253

[85] Mitra AK Zillhardt M Hua Y Tiwari P Murmann AE Peter ME. MicroRNAs reprogram normal fibroblasts into cancer-associated fibroblasts in ovarian cancer. Cancer Discov. (2012) 2:1100–8. 10.1158/2159-8290.CD-12-0206 23171795 PMC3685866

[86] Bullock MD Pickard KM Nielsen BS Sayan AE Jenei V Mellone M. Pleiotropic actions of miR-21 highlight the critical role of deregulated stromal microRNAs during colorectal cancer progression. Cell Death Dis. (2013) 4:e684. 10.1038/cddis.2013.213 23788041 PMC3702298

[87] Naito Y Sakamoto N Oue N Yashiro M Sentani K Yanagihara K. MicroRNA-143 regulates collagen type III expression in stromal fibroblasts of scirrhous type gastric cancer. Cancer Sci. (2014) 105:228–35. 10.1111/cas.12329 24283360 PMC4317817

[88] Wang Y Gu J Roth JA Hildebrandt MA Lippman SM Ye Y. Pathway-based serum microRNA profiling and survival in patients with advanced stage non-small cell lung cancer. Cancer Res. (2013) 73:4801–9. 10.1158/0008-5472.CAN-12-3273 23774211 PMC3760306

[89] Dougan M Dranoff G. Immune therapy for cancer. Annu Rev Immunol. (2009) 27:83–117. 10.1146/annurev.immunol.021908.132544 19007331

[90] Verma IM Weitzman MD. Gene therapy: twenty-first century medicine. Annu Rev Biochem. (2005) 74:711–38. 10.1146/annurev.biochem.74.050304.091637 15952901

[91] Bader AG. miR-34 - a microRNA replacement therapy is headed to the clinic. Front Genet. (2012) 3:120. 10.3389/fgene.2012.00120 22783274 PMC3387671

[92] Qian X Long L Shi Z Liu C Qiu M Sheng J. Star-branched amphiphilic PLA-b-PDMAEMA copolymers for co-delivery of miR-21 inhibitor and doxorubicin to treat glioma. Biomaterials (2014) 35:2322–35. 10.1016/j.biomaterials.2013.11.039 24332459

[93] Chakraborty C Sharma AR Sharma G Doss CGP Lee SS. Therapeutic miRNA and siRNA: moving from bench to clinic as next generation medicine. Mol Ther Nucleic Acids (2017) 8:132–43. 10.1016/j.omtn.2017.06.005 28918016 PMC5496203

[94] Bouchie A. First microRNA mimic enters clinic. Nat Biotechnol. (2013) 31:577. 10.1038/nbt0713-577 23839128

